# Influence of Autologous Bone Marrow Stem Cell Therapy on the Levels of Inflammatory Factors and Conexin43 of Patients with Moyamoya Disease

**DOI:** 10.1155/2022/7620287

**Published:** 2022-08-23

**Authors:** Liming Zhao, Tianxiao Li, Bingqian Xue, Hao Liang, Shao Zhang, Ruiyu Wu, Gaochao Guo, Tao Gao, Yang Liu, Yuxue Sun, Chaoyue Li

**Affiliations:** ^1^Department of Neurosurgery, Zhengzhou University People's Hospital, Henan Provincial People's Hospital, Zhengzhou 450003, China; ^2^Department of Neurosurgery, Henan University People's Hospital, Henan Provincial People's Hospital, Zhengzhou 450003, China

## Abstract

Moyamoya disease is a medical condition that shows the typical characteristics like continuous and chronic thickening of the walls and the contraction of the internal carotid artery; as a result, the internal diameter of the artery gets narrowed. There are six phases of the disease ranging from I to VI (moyamoya vessels completely disappear, followed by the complete blockage of the arteries). Surgery is a commonly recommended treatment for the moyamoya disease. Our research study identifies the effect of autologous bone marrow stem cell therapy (ABMSCT) on the levels of inflammatory factors and Conexin43 (Cx43) protein in patients suffering from moyamoya. In our study, we have selected 52 moyamoya patients admitted to our hospital from 30 July 2019 to 10 February 2020. The control group (CG) was treated with superficial temporal artery to a middle cerebral artery (STA-MCA) bypass + encephalo-duro-myosinangiosis (EDMS). The experimental group (Exp. Grp) was treated with ABMSC. The cerebral vascular tissue of the patients was treated with hematoxylin-eosin (HE) staining. Immunohistochemical staining was used to identify the levels of Cx43 protein. The concentrations of vascular endothelial growth factor (VEGF), inflammatory factor interleukin-6 (IL6), interleukin-1*β* (IL1*β*), tumor necrosis factor (TNF*α*), and anti-inflammatory factor interleukin-1*β* (IL1*β*) were determined by enzyme-linked immunosorbent assay (ELISA). We have found that after treatment of the expression of Cx43 protein, the proportions of grade IV (7.7%), grade III (311.5%), and grade II (3.8%) patients in the Exp. Grp were lower than those in the CG. The proportion of grade I patients in the Exp. Grp (77%) was higher than that in the CG (38.5%). After treatment, the inflammatory factors IL6 (0.97 ± 0.82 pg/mL), IL1*β* (8.33 ± 1.21 pg/mL), and TNF*α* (1.73 ± 0.71 pg/mL) in the Exp. Grp were lower than those in the CG. The anti-inflammatory factor IL1*β* (15.09 ± 4.72 pg/mL) increased in the Exp. Grp compared with the CG (11.25 ± 3.48 pg/mL) post treatment. Intracranial infection, hydrocephalus, hemiplegia, and transient neurological dysfunction in the Exp. Grp were lower than those in the CG, with statistical differences (*P* < 0.05). Our study suggests that the treatment of autologous bone marrow stem cells (ABMSC) was beneficial to balance the inflammatory response of disorders, reduce the damage of vascular tissue in the brain, and regulate tissue repair by co-acting with various inflammatory factors as compared to traditional surgery. We conclude that the involvement of Cx43 in the occurrence and development of moyamoya. We also have found that the risk factors of intracranial infection after ABMSCT were less as compared to those after conventional surgery.

## 1. Introduction

Moyamoya is a disease that comes in the rare category. It is related to the brain and its blood vessels with an unknown cause and a high mortality and disability rate. The incidence is about 4%, of which about 1.5% all have a family history [1, 2]. The main symptoms are narrowing or occlusion of bilateral internal arteries in the brain. Patients often have headaches, dizziness, epilepsy, aphasia, dyskinesia, and other uncomfortable symptoms, and the symptoms of some patients are not particularly obvious [3, 4]. With the improvement in modern medical levels, moyamoya can be treated early and gradually. However, due to the defects of repeated attacks and the long duration of moyamoya, the occurrence may cause patients to suffer from temporary or permanent neurological damage, cognitive impairment, and other major diseases, and in more severe cases, death [5, 6]. It seriously affects the daily life and economy of patients and their families, making them suffer from psychological torture. Therefore, early detection and effective treatment can greatly improve the prognosis of patients and it is of great significance.

In Reference [7], the authors present a medical evaluation of MMS and moyamoya. They assess the epidemiological, pathological, and historical background of moyamoya They also present the clinical and radiographical outcomes and investigative imaging modalities. They highlight the efficacy of medical and surgical treatment and discuss the problems associated with surgical treatment. In Reference [8], the authors focus on the vasculopathy related to MYH11 mutations. They also state the effect of MYH11 mutations on cerebral arteriopathy. It is recommended that MYH11 testing could be thought of in the case of children suffering from moyamoya. In Reference [1], the authors state that the bone-marrow stem mobilization after revascularization in sufferers of moyamoya can make the recovery process faster and encourage the development of new blood vessels. It is able to lessen infection and improve patients' overall quality of life. In Reference [9], the authors study the clinical performance of multipoint cranium drilling and the usage of simvastatin as the remedy for moyamoya. Treating moyamoya by this method is safe and efficient, as it may assist in healing of nervous functions and it also improves patients' everyday life skills and quality of lifestyle. In Reference [10], the authors investigate if moyamoya can be caused due to mechanical stress caused by the blood flow that opposes the vulnerable intracranial vasculature. They also additionally look at the possibility that the angle between the supraclionoid segments and cavernous can be the cause of the disorder. The moyamoya ailment may result from mechanical factors acting at the ICA bifurcation. According to the research study, the angle may get increased because of blood vessel wall factors. In Reference [11], the authors point out the efficacy of surgical revascularization in heading off stroke in moyamoya. Surgical revascularization consists of augmenting the intracranial blood flow with the use of an external carotid system by use of direct bypass. The pial synangiosis can also be used. In Reference [12], the authors state that the moyamoya disease is found more amongst the younger patients. The patients are vulnerable to perioperative ischemic complications. They also state that the recognition of moyamoya might also contribute to the better surgical results obtained through perioperative management based on suitable surgical risk stratification. In References [13, 14], the authors try to identify whether or not extracranial-intracranial bypass can enhance patient prognosis and lessen the prevalence of rebleeding. They conclude that direct bypass lessens the danger of frequently happening hemorrhages in moyamoya. In Reference [15], the Kaplan–Meier analysis is performed to reveal the difference between the nonsurgical and surgical groups. It also suggested the preventive effect of bypass on rebleeding.

In this research study, we look into the results of autologous bone-marrow stem cell therapy (ABMST) on the levels of inflammatory factors and Conexin43 (Cx43) protein in patients suffering with moyamoya. This research will serve as a guideline for the treatment of the disease.

The highlights of the research are given below:

1. Identification of the population for the study
2. Proposing the ABMSC treatment for moyamoya
3. Determining the concentrations of VEGF, inflammatory factors IL6, IL1*β*, TNF*α*, and anti-inflammatory factor IL1*β* in the serum
4. Cx43 immunohistochemical determination
5. Statistical analysis is performed using the *χ*^2^ test. For statistical processing, the *t*-test was used

The next sections of the paper elaborate the proposed work and results.

## 2. Materials and Methodology

The flow of the methodology is as follows:

1. *Information gathering*: we gathered the information from the selected group. The inclusion and exclusion criteria have been mentioned in the section given below.
2. *The treatment*: the CG (control group) was treated on the basis of conventional drug therapy, and the Exp. Grp was treated with ABMSC.
3. *Staining*: HE staining and Immunohistochemical staining was performed in order to observe the cell level detailing.
4. *ELISA*: It was used for the binding of antibodies to antigens. Cerebral vascular tissue samples were dissolved to determine the concentrations of VEGF, inflammatory factors IL6, IL1*β*, TNF*α*, and anti-inflammatory factor IL1*β* in the serum.
5. *Cx43 immunohistochemical positive determination*: Immunohistochemical staining is utilized to find out the levels of Cx43 protein.
6. *Analytical study*: In this study, SPSS13.0 was used for the analysis.

### 2.1. Basic Information and Grouping

52 cases of patients from our hospital from 30 July 2019 to 10 February 2020 were selected, including 24 males, 28 females, 8 children, and 44 adults, with an average age of 42.56 ± 11.78. Among them, 9 patients had a history of hypertension and heart disease; 14 presented with intracerebral hemorrhage; and 38 presented with ischemic symptoms. Suzuki staging: 29 cases in phase 3, 23 cases in phase 4.

#### 2.1.1. Inclusion Criteria

The inclusion criteria were as follows:

- Patients with ages ranging from 18–65  yrs
- Patients diagnosed with moyamoya disease/syndrome, and all patients were confirmed by DSA angiography
- Initial symptoms presented as cerebral ischemia symptoms, such as transient ischemic attacks (TIA), limb numbness, visual defect, intermittent headache, dizziness, asymptomatic, or stroke (older than 3 months)
- The admission's NIHSS score was less than 2
- The results of ECG and pulmonary function testing showed normal

#### 2.1.2. Exclusion Criteria

The exclusion criteria were as follows:

- DSA showed that moyamoya was complicated with other cerebrovascular ailments such as arteriovenous malformation (AVM), aneurysm, and posterior-circulation vascular disease.
- Patients had a previous history of cerebral parenchyma hemorrhage, subarachnoid, and intraventricular hemorrhage.
- Patients suffering from metabolic and endocrine system disorders.

52 moyamoya patients were divided into the CG and the Exp. Grp, each with 26 cases. The CG received conventional intracerebral and extracerebral vascular bypass surgery, while the Exp. Grp received the ABMSC.

### 2.2. The Treatment

In the CG, on the basis of conventional drug therapy, all patients underwent a combined revascularization procedure which included indirect flow augmentation by EDMS and direct bypass through the STA-MCA bypass as previously described elsewhere (Peter book). Prior to performing the anastomosis, all patients underwent indocyanine green (ICG) and FLOW800 analysis to ensure the anastomosis side was in a low perfusion area in correlation with the radiological findings [16, 17]. We decided to perform STA-MCA bypass in relation to the hypoperfusion extension. The blood vessels cut from the anastomotic site were used as samples. The Exp. Grp was treated with ABMSC on the basis of the CG. After surgery, 2.5 *μ*g/kg recombinant human granulocyte colony and granulocyte-macrophage colony-stimulating factors were alternately injected subcutaneously every three days for 21 consecutive days.

### 2.3. HE Staining

The frozen cerebral vascular tissue was fixed with a 10% solution of formalin. Gradient dehydration was carried out with ethanol from a low to a high degree. Paraffin embedding was performed for 4 *μ*m sections. The sections were routinely stained with HE, dewaxed with xylene, washed with ethanol at all levels, and then the staining was done for 10 min using hematoxylin and then rinsed out with tap water. After it was stained in eosin for 2 min, conventional ethanol was dehydrated, xylene was transparently sealed and fixed with neutral resin, and histopathological changes were examined by light microscopy. The nuclei were dark blue, and the cytoplasm and fibrous tissues were red in varying shades. The sections with better staining were photographed and analyzed.

### 2.4. Immunohistochemical Staining

Immunohistochemical staining is a widely used technique used to identify the antigens present in biological tissue samples [18]. The vascular tissue in the brain was removed, and the sections were embedded by dehydration. Then, immunohistochemical staining was performed. The section was dehydrated and hydrated first. Antigen repair was performed to block endogenous peroxidase. It was washed with phosphoric acid buffer (PBS), and then goat serum blocking solution was added. Diluted primary antibody and biotinylated secondary antibody against goat anti-rabbit IgG antibody were added. The slides were washed for three times for 5 mins each, using PBS. Then, diaminobenzidine (DAB) was added for color development, the nuclei were re-stained with hematoxylin for 4 min, the cells were dehydrated with gradient ethanol, and the tablets were sealed with xylene transparent neutral resin. It was shot under a microscope to observe the vascular smooth muscle cell cytoplasmic expression of *α*-SMA. The positive color was tan, and the nuclei were re-stained light blue. The results of Cx43 immunohistochemical staining showed that the tan color was positive.

### 2.5. Enzyme-Linked Immunosorbent Assay (ELISA)

ELISA is used to measure antibodies, antigens, proteins, and glycoproteins in biological samples [19]. Cerebral vascular tissue samples were dissolved to determine the concentrations of VEGF, inflammatory factors IL6, IL1*β*, TNF*α*, and anti-inflammatory factor IL1*β* in the serum. The samples were incubated with the standard in the reaction well, and then a biotin-labeled primary antibody was added and it was incubated at room temperature for 2 h. After the reaction, the product was washed with buffer solution many times. The color solution 3,3′,5,5′-tetramethylbenzidine (TMB) was then added to each of the wells and it was incubated (at room temperature) away from the light. After the termination solution was added, the value of optical density (OD) was determined by an enzyme marker at a wavelength of 540 nm. The sample concentration was identified in accordance with the standard curve of the sample, and the actual concentration of the sample was obtained by multiplying the dilution factor.

### 2.6. Cx43 Immunohistochemical Positive Determination

Conexin43 (Cx43) is the intercellular gap junction protein. It is responsible for the normal function of arteries. It also plays an important role in the development of cardiovascular ailments. According to the positive cell percentage in the immunohistochemical results in the same kind of cells and the staining intensity of positive cells, the section results can be classified into grades I–IV by semiquantitative scoring. At the IV level, the positive cells accounted for more than 50%, and the positive cells were tan in color. At the III level, the positive cell percentage was 25%–50%, and the positive cells were brownish. At the II level, the positive cell percentage was 10%–25%, and the positive cells were yellowish in color. At the I level, the positive cell's percentage was less than 10%, and they were not stained.

### 2.7. Statistical Analysis

In this study, SPSS13.0 was used to analyze and process all experimental data. The counting data was represented by a percentage (%). A *χ*^2^ test was performed. The data were expressed as mean ± standard deviation (*X* ± *s*), and the *t* test was used for statistical processing. *P* < 0.05 indicated the differences that were statistically significant.

## 3. Results

### 3.1. Comparison of Age of Study Subjects

**Figure 1:**
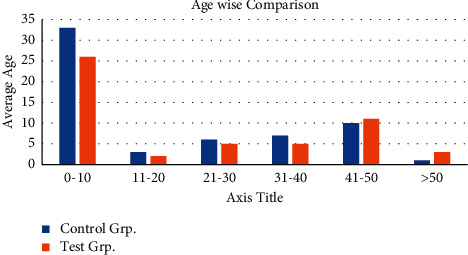
Comparison of the average age of the patients and the proportion of the population in each age group in the CG and the experimental control.

The mean age of the patients in the CG and the Exp. Grp and the ages of less than 19, 20–30, 31–40, 41–50, and more than 51 were compared and analyzed, as shown in Figure 1. The average age of the CG was 33.11 ± 10.02, and that of the Exp. Grp was 28.76 ± 11.8. The proportions of patients in the CG and the Exp. Grp aged less than 19, 20–30, 31–40, 41–50, and over 51 were (7.7%, 23.1%, 26.9%, 38.5%, and 3.8%) and (11.5%, 19.2%, 15.4%, 42.3%, and 11.5%), respectively. A statistical difference (stat. difference) was not found in the mean age and proportion of each age group between the CG and the Exp. Grp (*P* > 0.05). The following figure (Figure 1) shows the comparison of the average age of the patients and the proportion of the population in each age group in the CG and the experimental control.

### 3.2. MMD Diagnosed by DSA (Digital Subtraction Angiography)

**Figure 2:**
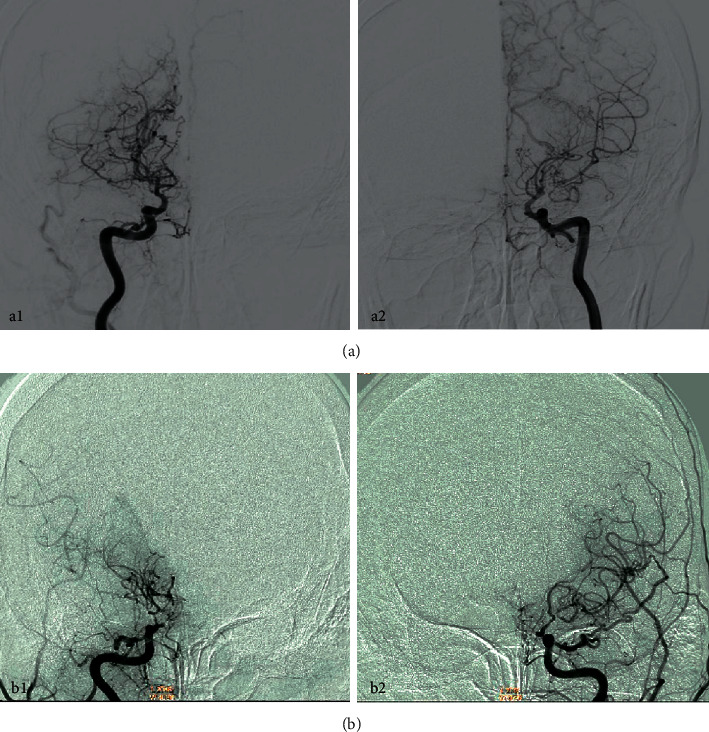
DSA images obtained in two moyamoya patients (a) a 9-year-old, female and (b) an adult, 35 years old male; the blue arrow pointed to the lesion area.

A DSA was performed on two moyamoya patients, and the results are shown in Figure 2.  Figure 2(a) shows a 9-year-old child, female. The DSA image of the child showed bilateral middle cerebral arteries, occlusion of the anterior cerebral artery, and multiple tortuous collateral circulation vessels inside the skull.  Figure 2(b) shows the DSA image of a 35-years-old, male adult suffering from moyamoya who showed a sudden cerebral infarction. The DSA showed occlusion of the middle cerebral artery and left internal carotid artery. It also shows the multiple localized stenosis of the right middle cerebral artery. The following figure (Figure 2) shows the DSA images obtained in two moyamoya patients.

### 3.3. HE Staining of Cerebral Blood Vessels in Moyamoya Patients

**Figure 3:**
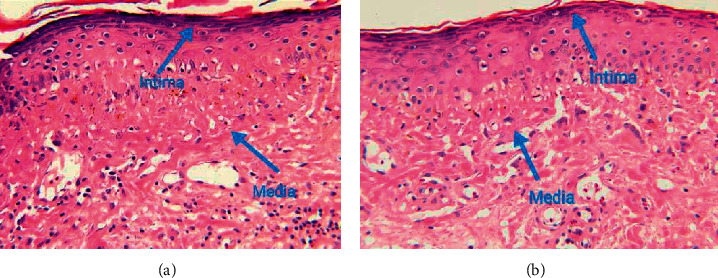
HE staining of cerebral blood vessels in moyamoya patients. (a) CG (control group) and (b) Exp. Grp. The blue arrows point to the inner and middle membranes of the vessels.

HE staining of cerebral blood vessels in moyamoya patients in the Exp. Grp and the CG are shown in Figure 3. The inner and middle membranes were thickened to different degrees.  Figure 3(a) depicts severe thickening of the vascular intima with cells passing through the elastic membrane rupture.  Figure 3(a) depicts moderate thickening of the intima, thinning of the medium-membrane, and interconnections between the intima and medium-membrane cells. Figure 3 shows the HE staining of cerebral blood vessels in moyamoya patients.

### 3.4. Cx43 Protein Expression and Immunohistochemical Staining Results

**Figure 4:**
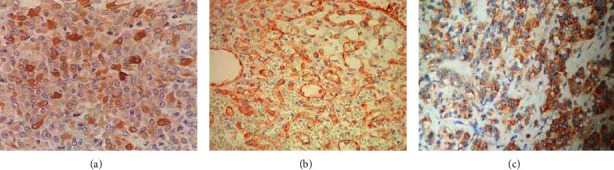
Immunohistochemical staining of the Cx43 protein expression. (a) Positive expression (Positive Exp.) of grade II Cx43 cells; (b) positive Exp. of grade III Cx43 cells; and (c) positive Exp. of grade IV Cx43 cells.

Immunohistochemical results of the Cx43 protein expression in the CG and the Exp. Grp after treatment are shown in Figure 4. In the CG, Cx43 protein in the cerebral blood vessels was mainly strongly positive or positively expressed, and the color was dark brown. Cx43 protein was not expressed or was only weakly positive in the Exp. Grp, and the color was light yellow. Figure 4 shows the immunohistochemical staining of the Cx43 protein expression.

**Figure 5:**
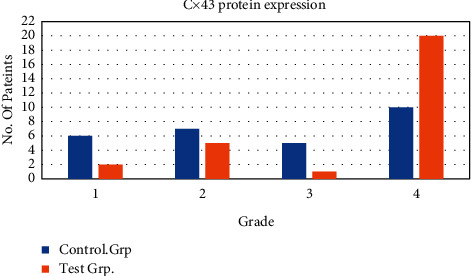
Cx43 protein expression in the CG and the Exp. Grp after treatment. ^*∗*^indicated that there was a statistical difference between the groups *P* < 0.05.

The expression of Cx43 protein in the CG (26 cases) and the Exp. Grp (26 cases) after treatment were analyzed as shown in Figure 5. The Cx43 positive grading index showed that the proportion of grade IV patients (2 cases were 7.7%), grade III patients (3 cases were 11.5%), and grade II patients (1 case was 3.8%) in the Exp. Grp was lower than that of grade IV patients (6 cases were 23.1%), grade III patients (7 cases were 26.9%), and grade II patients (3 cases were 11.5%) in the CG. The proportion of grade I patients in the Exp. Grp (77% in 20 cases) was higher than that in the CG (38.5% in 10 cases), and there was a statistical difference between the two groups (*P* < 0.05). Figure 5 shows the Cx43 protein expression in CG and Exp. Grp after treatment.

### 3.5. ELISA Results of VEGF in CG (Control Group) and Exp. Grp

**Figure 6:**
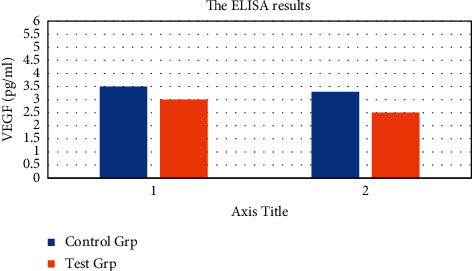
The ELISA results' comparison of VEGF before and after treatment between the CG and the Exp. Grp. ^*∗*^indicates that after treatment, there was a ST between the CG and the Exp. Grp, *P* < 0.05. (a) before treatment and (b) after treatment.

The results of VEGF ELISA before and after treatment in the CG and the Exp. Grp were compared and analyzed as shown in Figure 6. There was no significant difference in VEGF (3.44 ± 1.97 pg/mL) between the Exp. Grp and the CG (3.57 ± 2.09 pg/mL) (*P* > 0.05) before treatment. After treatment, the Exp. Grp (2.46 ± 2.11 pg/mL) was less than the CG (3.01 ± 1.54 pg/mL), and there was a stat. difference (*P* < 0.05). Figure 6 shows the ELISA results' comparison of VEGF before and after treatment between the CG and the Exp. Grp.

### 3.6. The ELISA Results Obtained from the CG and the Exp. Grp

**Figure 7:**
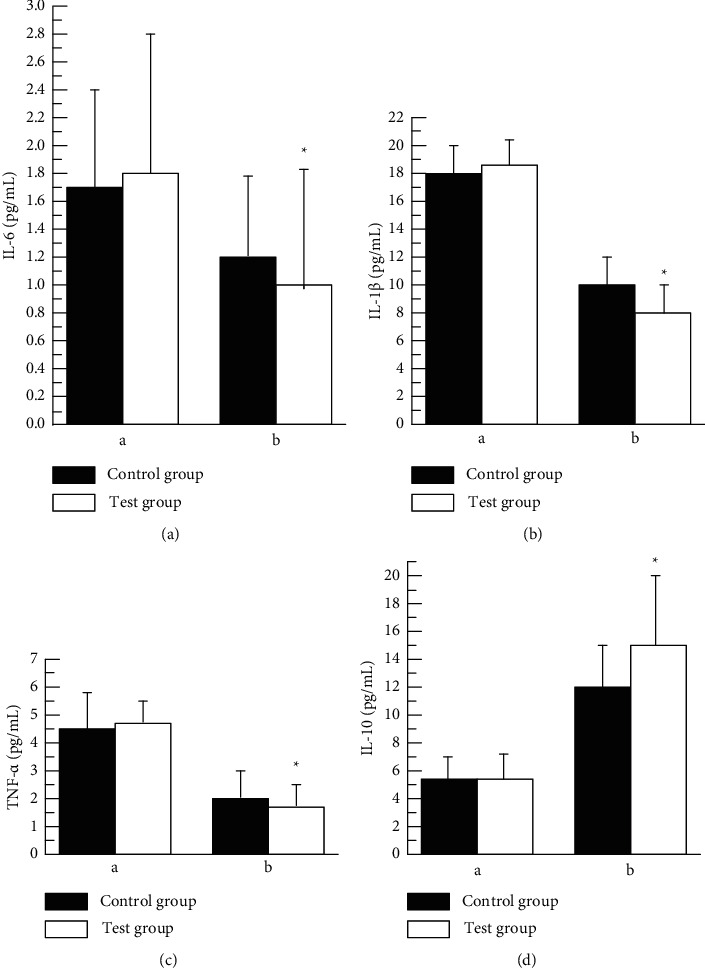
The ELISA results of related inflammatory factors in CG and the Exp. Grp before and after treatment ((a) IL6, (b) IL1*β*, (c) TNF*α*, and (d) IL1*β*). ^*∗*^indicates that after treatment, there was a ST between the CG and the Exp. Grp, *P* < 0.05 (a) before treatment and (b) after treatment.

The ELISA results of inflammatory factors IL6, IL1*β*, and TNF*α* were compared before and after treatment in the CG and the Exp. Grp. As shown in Figures 7(a)–7(c), the inflammatory factors IL6, IL1*β*, and TNF*α* in the Exp. Grp before treatment showed no ST as compared with the CG (*P* > 0.05). After treatment, the inflammatory factor IL6 (0.97 ± 0.82 pg/mL) in the Exp. Grp was lower than that in the CG (1.24 ± 0.45 pg/mL), and the inflammatory factor IL1*β* in the Exp. Grp (8.33 ± 1.21 pg/mL) was lower than that in the CG (10.13 ± 1.54 pg/mL), and the inflammatory factor TNF*α* (1.73 ± 0.71 pg/mL) in the Exp. Grp was lower than that in the CG (2.01 ± 1.03 pg/mL). After treatment, the inflammatory factors IL6, IL1*β*, and TNF*α* in the Exp. Grp were statistically (ST) different from those in the CG (*P* < 0.05).

The ELISA results of the anti-inflammatory factor IL1*β* before and after treatment were compared between the CG and the Exp. Grp. As shown in Figure 7(d), there was no ST in the anti-inflammatory factor IL1*β* (5.58 ± 1.34 pg/mL) in the Exp. Grp compared with the CG (5.62 ± 1.01 pg/mL) before treatment (*P* > 0.05). After treatment, the anti-inflammatory factor IL1*β* (15.09 ± 4.72 pg/mL) increased in the Exp. Grp compared with the CG (11.25 ± 3.48 pg/mL), showing an ST with *P* < 0.05. The following figure (Figure 6) shows the ELISA results of related inflammatory factors in the control group (CG) and the experimental group (EG) before and after treatment (A-IL6, B-IL1*β*, C-TNF*α*, and D-IL1*β*)

### 3.7. Complications after Treatment in the CG and the Exp. Grp

**Figure 8:**
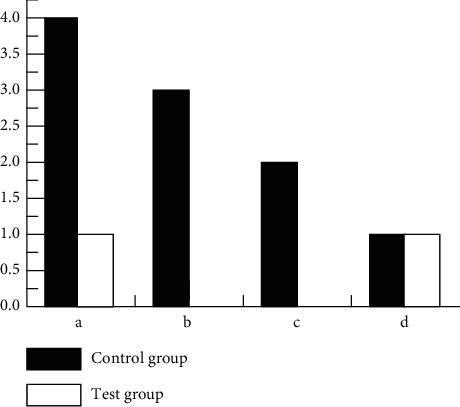
Complications of (a) intracranial infection, (b) hydrocephalus, (c) hemiplegia, and (d) transient neurological dysfunction in the CG and the Exp. Grp after treatment. ^*∗*^indicates that there was a ST between the CG and the Exp. Grp, *P* < 0.05.

Complications such as intracranial infection, hydrocephalus, hemiplegia, and transient neurological dysfunction were analyzed in the CG and the Exp. Grp after treatment as shown in Figure 8. After treatment, intracranial infection (1 case 3.8%), hydrocephalus (0 cases), hemiplegia (0 cases), and transient neurological dysfunction (1 case 3.8%) were lower in the Exp. Grp than in the CG with intracranial infection (4 cases 15.4%), hydrocephalus (3 cases 11.5%), hemiplegia (2 cases 7.7%), and transient neurological dysfunction (1 case 3.8%). There was a ST between the two (*P* < 0.05).

## 4. Discussion

Moyamoya seriously affects the mental as well as the physical health of children and adolescents and is the main cause of cerebrovascular accidents in this population. In recent years, it has gradually attracted the attention of scholars from all over the world, but there is no effective method to control the occurrence and development of Moyamoya. In this study, 52 cases of moyamoya patients were selected for relevant research. Histopathological examination of the patient's intracranial blood vessels revealed rupture of the internal elastic membrane and uneven thickening of the vascular intima. It was found that there were endometrial cells and mesenchymal cells in the rupture of the internal elastic membrane, and the mesenchymal membrane was significantly thinner, which was consistent with mmd-related pathological changes and consistent with domestic and foreign literature. By comparing the expression of Cx43 protein in the CG and the Exp. Grp after treatment, the immunohistochemical results showed the proportions of grade IV patients (7.7%), grade III patients (311.5%), and grade II patients (3.8%) in the Exp. Grp were lower than that of grade IV patients (23.1%), grade III patients (26.9%), and grade II patients (11.5%) in the CG. The proportion of grade I patients in the Exp. Grp (77%) was higher than that in the CG (38.5%), and there was a statistical difference between the experimented groups (*P* < 0.05). Cx43 protein was not expressed or weakly positive in the middle and inner membranes of the blood vessels in the Exp. Grp, while Cx43 protein was strongly or positively expressed in the middle and inner membranes of the blood vessels in the CG. It was speculated that the Cx43 protein was involved in the migration as well as the proliferation of moyamoya in smooth muscle cells. VSMC or phenotypic transformation of VSMC; therefore, the Cx43 protein may be the cause of the development of moyamoya.

In this study, ELISA results of VEGF, inflammatory factors IL6, IL1*β*, TNF*α*, and anti-inflammatory factor IL1*β* were compared before and after treatment in the CG and the Exp. Grp. After treatment, the Exp. Grp (2.46 ± 2.11 pg/mL) was lower than the CG (3.01 ± 1.54 pg/mL), showing a statistical difference (ST) with *P* < 0.05. In the pathological state, VEGF stimulated the formation and growth of tumor blood vessels and caused tissue edema in the inflammatory state. The increase of VEGF was also related to vascular diseases and played a certain role in the formation of moyamoya reticular blood vessels, which was in tandem with the results of Jeon [18]. After treatment, the inflammatory factors IL6 (0.97 ± 0.82 pg/mL), IL1*β* (8.33 ± 1.21 pg/mL), and TNF*α* (1.73 ± 0.71 pg/mL) in the Exp. Grp were lower than those in the CG. After treatment, the anti-inflammatory factor IL1*β* (15.09 ± 4.72 pg/mL) in the Exp. Grp increased compared with that in the CG(11.25 ± 3.48 pg/mL), and the difference was statistically significant (*P* < 0.05). After ABMSCT in moyamoya patients, the inflammatory factors IL6, IL1*β*, and TNF*α* in plasma can be reduced, and the anti-inflammatory factor IL1*β* can be increased. It indicated that ABMSC has an intervention effect on the secretion of various inflammatory factors of moyamoya, thus providing a repairable microenvironment for the vascular tissues in the brain and playing a protective role on the tissues [17].

The analysis of the prognostic complications showed that the intracranial infection, hydrocephalus, hemiplegia, and transient neurological dysfunction after treatment in the Exp. Grp were lower than those in the CG, and there was a statistical difference between the two (*P* < 0.05), which indicated that the prognosis of ABMSC for moyamoya was better, and the physiological indicators of the patients have been significantly improved, and the adverse reactions of the patients have not been increased.

## 5. Conclusion

In our study, the effects of ABMSCT on the levels of inflammatory factors and Cx43 protein in patients with moyamoya have been analyzed. The results show the basic pathological changes of moyamoya patients resulted in an uneven thickening of the cerebral vascular intima. It is concluded that the involvement of Cx43 in the occurrence and development of moyamoya is significant. It can also be summarized that the treatment of ABMSC is beneficial to balance the inflammatory response of the disorder, reduce the damage of vascular tissue in the brain, and regulate the tissue repair by co-acting with various inflammatory factors, which provided certain guidelines for the clinical treatment of moyamoya. It also investigated the risk factors of intracranial infection after autologous bone-marrow stem cell therapy. It has been found that the rates of intracranial infection, hydrocephalus, hemiplegia, and transient neurological dysfunction after treatment in the patients treated with ABMSC are lower than in those patients who were treated on the basis of conventional drug therapy. Our future progress will include follow-up analysis of the prognosis of patients after autologous bone marrow stem cell treatment, which can be further confirmed by the data.

## Data Availability

The data can be made available on request.
